# Protocol for a randomized controlled trial to assess two procedures of vaginal native tissue repair for pelvic organ prolapse at the time of the questioning on vaginal prosthesis: the TAPP trial

**DOI:** 10.1186/s13063-020-04512-x

**Published:** 2020-07-08

**Authors:** A. Lacorre, F. Vidal, S. Campagne-Loiseau, B. Marin, Y. Aubard, F. Siegerth, C. Mesnard, E. Chantalat, C. Hocke, T. Gauthier

**Affiliations:** 1 Department of Gynecology and Obstetrics, CHU Limoges, 8 avenue Dominique Larrey, 87042 Limoges Cedex, France; 2 Department of Gynecology and Obstetrics, Hôpital de Guéret, 8 avenue Dominique Larrey, 87042 Limoges Cedex, France; 3 Pôle Femme Mère Couple, Hôpital Paule de Viguier, CHU Purpan, 330 avenue de Grande Bretagne, 31059 Toulouse, France; 4 Department of Gynecology and Obstetrics, CHU Clermont-Ferrand Estaing, 1 place Lucie Aubrac, 63100 Clermont-Ferrand, France; 5 Institute of Neurological Epidemiology and Tropical Neurology, Faculté de Médecine de Limoges, 2 rue du Docteur Marcland, 87025 Limoges, France; 6 Department of Gynecology and Obstetrics, Hôpital de Tulle, 3 place Maschat, 19000 Tulle, France; 7 Department of Gynecology and Obstetrics, Hôpital de Brive-La-Gaillarde, 3 boulevard Dr Verlhac, 19100 Brive-La-Gaillarde, France; 8 Department of Gynecology and Obstetrics, CHU Toulouse Rangueil, 1 avenue du Professeur Jean Poulhès, 31400 Toulouse, France; 9 Department of Gynecology and Obstetrics, CHU Bordeaux Pellegrin, Centre Aliénor d’Aquitaine, Place Amélie Raba Léon, 33076 Bordeaux, France

**Keywords:** Anterior colporraphy, Vaginal patch plastron, Cystocele, Pelvic organ prolapse, Randomized controlled trial, Surgery, Combined definition of success

## Abstract

**Background:**

Native tissue cystocele repair has been the cornerstone of prolapse surgery, especially since the learned societies warned clinicians and patients about serious mesh-related complications. Surgical techniques mainly consist in anterior colporraphy and vaginal patch plastron. However, success rates of native tissue cystocele repair are heterogeneous, depending on the design of studies and definition of outcomes. To date, high-quality data comparing vaginal native tissue procedures are still lacking.

**Methods:**

Herein we aimed to describe the design of the first randomized controlled trial (TAPP) comparing anterior colporraphy (plication of the muscularis and adventitial layers of the vaginal wall) and vaginal patch plastron (bladder support anchored on the tendinous arch of the pelvic fascia by lateral sutures) techniques.

Our aim is to assess the effectiveness of vaginal native tissue repair at 1 year for cystocele with a combined definition of success—anatomic and functional. The primary endpoint will be the success rate 1 year after surgery with a composite of objective and subjective measures (Aa and Ba points < 0 from POP-Q (Pelvic Organ Prolapse Quantification System) and a negative answer to question 3 of Pelvic Floor Distress Inventory and no need for additional treatment).

**Discussion:**

A prospective study has found a success rate at 35% for anterior colporraphy based on a combined definition, both anatomic and functional, as recently recommended. However, the definition of anatomic was strict (POP-Q< 2), while it seems that the best definition of anatomic success is “no prolapse among the hymen”, that is to say Aa and Ba points from the POP-Q classification < 0. We hypothesize that vaginal patch plastron will have a better anatomic and functional success comparatively to anterior colporraphy because native tissue is added, as it corrects both median and lateral cystoceles thanks to bilateral paravaginal suspension.

**Trial registration:**

*CHU LIMOGES* is the sponsor of this research (n°87RI18_0013).

This research is supported by the French Department of Health (PHRC 2018-A03476-49) and will be conducted with the support of DGOS (PHRC interregional – GIRCI SOHO).

The study protocol was approved by the Human Subjects Protection Review Board (Comité de Protection des Personnes) on May 16, 2019.

The trial is registered in the ClinicalTrials.gov registry (NCT03875989).

## Background

Pelvic organ prolapse (POP) is usually the result of loss of pelvic support. It is widely accepted that 50% of women after 50 years of age will develop prolapse, evaluated through the POP-Q classification (Pelvic Organ Prolapse Quantification). POP is associated with significant psychological distress and negatively affects quality of life. Cystocele is the main indication for POP surgery (67.7%) [1]. Native tissue cystocele repair has been the cornerstone of prolapse surgery especially since the learned societies (Food and Drug Administration (FDA), Haute Autorité de Santé (HAS), Collège National des Gynécologues-Obstétriciens Français (CNGOF)) [2–4] warned clinicians and patients about serious complications associated with transvaginal meshes. In France, 47.5% of vaginal cystocele repair is based on native tissue repair [5]. Anterior colporraphy and vaginal patch plastron are the most common procedures, with re-intervention rates lower than 4% at 1 year [6]. However, success rates of native tissue repair are heterogeneous (34.5% to 89%), depending on study design and the definition of outcomes. Furthermore, randomized trials comparing the effectiveness of vaginal native tissue techniques are still lacking. Hence, the choice of the surgical procedure is still not evidence-based and mostly subjective [1].

Cystocele is commonly treated by transvaginal repair with native tissue repair [5]. Sacrocolpopexy using synthetic mesh by laparoscopy is considered the surgical gold standard, but this procedure has several contraindications: baseline risk factors for mesh erosion (obesity, smoking, association with hysterectomy) and history of abdominal surgery [7]. The use of the vaginal surgery, considered as minimally invasive one, may be used in first intention for any patient suffering from symptomatic anterior POP [5]. While transvaginal mesh procedures have been largely studied, current evidence does not support their use as a first-line intervention for anterior compartment prolapse according the CNGOF, HAS, and FDA due to significant post-operative morbidity [2–4, 8].

### Anterior colporraphy

Anterior colporraphy is the most common procedure for cystocele repair. It involves a plication of the muscularis and adventitial layers of the vaginal wall. Anterior colporraphy is straightforward and relatively unchanged from its initial description over a century ago [9]. Anterior colporraphy alone has a reported success rate of only 38% when limited to the use of native tissue, but suture type and placement may improve success rates [10].

Prospective studies have shown anatomical success rates of anterior colporraphy ranging from 42% [11] to 69% [12]. Retrospective studies have shown functional success rates between 72.7% [5] and 78% [13].

A prospective comparative study reported a success rate of 35% for anterior colporraphy based on a combined anatomical and functional definition as recommended recently [6]. However, their definition of anatomic success was restrictive (POP-Q< 2), while “no prolapse among the hymen” (corresponding to Aa and Ba points < 0) may be a better definition of anatomical success. Iyer showed lower failure rates of anterior colporraphy with about 10% difference between anatomical failure defined by Aa point or Ba point greater than or equal to − 1 at 1 year post-operative and anterior failure as Aa or Ba of 0 [12].

### Vaginal patch plastron

Vaginal patch plastron was described in 1998 as a new surgical technique for the treatment of cystocele via the vaginal route. The technique is based on bladder support by a vaginal strip, isolated from the anterior colpocele, left attached to the bladder, and anchored on the tendinous arch of the pelvic fascia by six lateral sutures (three on each side of the plastron) [14].

In the prospective study of Chen et al., it was associated with a 74% functional success rate and a 55% anatomical success rate [15].

The use of native tissues, whose quality may be imperfect or deteriorate with time, exposes to the risk of recurrence. However, in case of vaginal patch plastron, the vagina left in contact with the bladder is a material of much better quality than colporraphy alone. The multiplication of native tissues, generating post-operative fibrosis, associated with anchorage on a strong ligamentous structure, allows to expect better outcomes compared to anterior colporraphy [16]. Indeed, vaginal plastron corrects median cystoceles with a vaginal strip as well as lateral cystoceles thanks to bilateral paravaginal suspension.

The aim of this study was thus to compare the effectiveness of the vaginal patch plastron versus anterior colporraphy 1 year after POP surgery, using a combined definition of success based on both anatomical and functional parameters [17].

## Methods/design

**Fig. 1:**
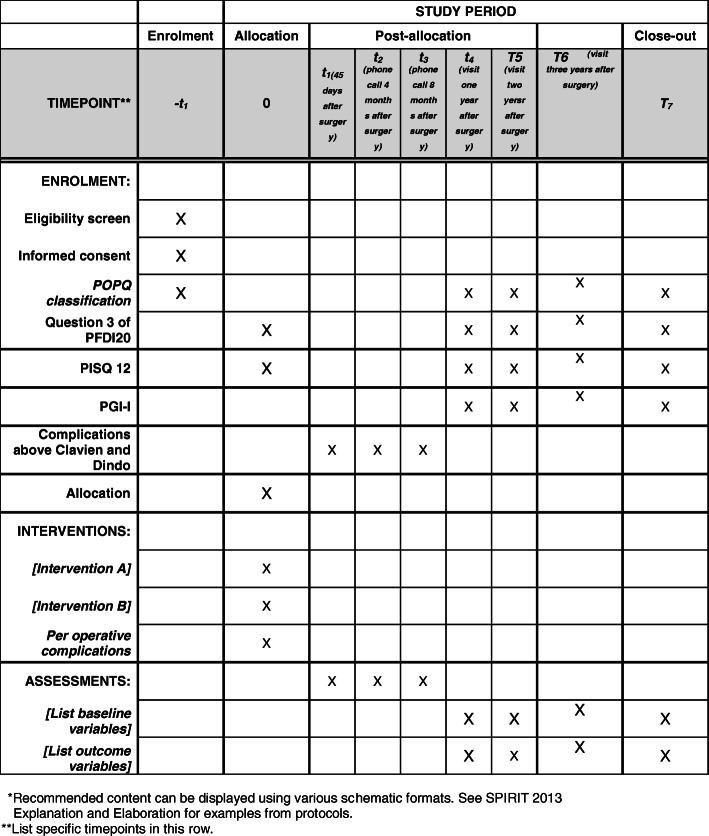
Example template of recommended content for the schedule of enrolment, interventions, and assessments. Recommended content can be displayed using various schematic formats. See SPIRIT 2013 Explanation and Elaboration for examples from protocols. **List specific timepoints in this row

Herein we introduce an experimental, multicenter (eight centers) parallel-group randomized controlled trial (1:1) to assess the effectiveness of the vaginal patch plastron at 1 year post-operatively in comparison with anterior colporraphy through a combined definition of success—anatomical and functional (Fig. 1).

## Results

The primary outcome will be assessed 1 year after surgery by an independent assessor blinded to the allocated treatment arm. It is defined as the success rate of POP surgery defined by a composite of objective and subjective parameters:

- Anatomical success defined by Aa and Ba values < 0 in the POP-Q system

AND

- Subjective success through reliable condition-specific quality-of-life questionnaires:
  ○ A negative answer to the question “Do you usually have a bulge or something falling out that you can see or feel in your vaginal area?” (question 3 of the PFDI-20)

AND

- ○ Range score of PGI-I 1 or 2 [18]

AND

- No need for re-intervention (medical or surgical) for recurrence of cystocele.

Secondary outcomes will comprise:

1. The failure rate of POP surgery defined by a composite of objective and subjective measures evaluated 1 year after surgery:

- ○ Recurrent prolapse defined by Aa and/or Ba values ≥0 in POP-Q system

OR

- ○ Subjective failure through reliable condition-specific quality-of-life questionnaires:
- ▪ A positive answer to the question “Do you usually have a bulge or something falling out that you can see or feel in your vaginal area?” (question 3 of the PFDI-20)

OR

- ▪ A PGI-I score > 2

OR

- Need for re-intervention (medical or surgical) for recurrence of cystocele. This secondary outcome will be assessed 1 year after surgery by an independent assessor blinded to the allocated arm.

2) Rate of post-operative complications according to the Clavien-Dindo classification 45 days after surgery, reported by the patient’s surgeon (not blinded).
3) Sexual function improvement will be evaluated by the difference in PISQ 12 score [19] (condition-specific quality-of-life questionnaire) between the inclusion and 1 year after surgery for sexually active women.
4) The failure rate of POP surgery defined by a composite of objective and subjective measures evaluated 2 years after surgery.
5) The failure rate of POP surgery defined by a composite of objective and subjective measures evaluated 3 years after surgery.

This secondary outcome will be evaluated 1, 2, and 3 years after surgery by an independent assessor blinded to the allocated arm.

The primary outcome and several secondary outcomes (items 1 and 3) will be evaluated according to a double-blind protocol (since the patient and his assessor will not be aware of the randomization arm that will have been assigned to the patient).

Comparisons will be made between the two groups of randomization in an intention-to-treat analysis. The inclusion period will extend over 30 months. The duration of follow-up for each patient will be 42 months. The trial will last 72 months.

### Study design

The study will be introduced to all women referred to participating centers for surgical repair of cystocele at the time of their pre-operative visit (screening visit). Patients will be definitively enrolled after giving their informed written consent.

### Randomization

Patients will be randomly assigned in a 1:1 ratio in the operating room to undergo anterior colporraphy (reference treatment) or vaginal patch plastron (experimental treatment) by a remote web-based randomization system.

In case of a computer problem, a paper randomization list will be available.

Since there is no specific contraindication for any of the two techniques, both surgical treatments can be performed in all patients included in the study. Hence, patients will not be aware of randomization assignment. In contrast, masking for the surgeon with respect to treatment allocated by randomization will not be feasible.

Hysterectomy will not be performed systematically; however, in case of hysterectomy, a sacrospinous ligament fixation or a high McCall culdoplasty will be performed.

The randomization will be stratified according to the center of care and to concomitant treatment of apical prolapse.

In this trial, we aim to compare the effectiveness of vaginal patch plastron versus anterior colporraphy at 1 year after surgery in patients with symptomatic cystocele. Therefore, both procedures will be standardized for all the surgeons in all participating centers. Standardization will be made through broadcasting video of surgical techniques and by performing, if necessary, double-team surgeries during implementation of the protocol.

#### Experimental arm A: vaginal patch plastron

We will delimitate a rectangular vaginal strip which will be isolated from the anterior colpocele. The upper edge of the strip is placed 2 cm from the urethral orifice. After lateral vesico-vaginal dissection, the paravesical fossae will be opened wide to highlight the tendinous arches. The vaginal plastron will be tied to the tendinous arch of the pelvic fascia by three lateral stitches (anterior/ lateral/ posterior) on each side of the plastron. Then the plastron will be tensioned and the cystocele will be suspended. Vaginal wall closure will end the procedure.

#### Active control arm B: anterior colporraphy

We will make a midline incision of the anterior vaginal wall from the urethrovesical junction to the vaginal apex or anterior fornix. The vaginal epithelium will be separated from the underlying fibromuscular layer (Halban fascia) after the midline incision. Midline plication of the fibromuscular layer will be obtained by interrupted horizontal stiches. Vaginal wall closure will end the procedure.

#### Approved associated surgical procedures

Approved associated surgical procedures comprise total hysterectomy with sacrospinous fixation or a high McCall culdoplasty, stress urinary incontinence cure, and rectocele repair by plication of the pre-rectal fascia.

In case of indication of associated hysterectomy or of prolapse of the vaginal fundus, a sacrospinofixation of Richter or a high McCall culdoplasty will be performed. In contrast, myorraphy of levator ani muscles cannot be conducted at the time of cystocele repair because of the higher rates of post-operative pain and dyspareunia associated with such a procedure.

Patients’ follow-up will comprise:

- A visit 45 days (± 10 days) after surgery to evaluate the post-operative complications according to the Clavien-Dindo classification (referent surgeon);
- Two additional visits at 4 and 8 months (± 1 week) after surgery to make sure they have not suffered from delayed post-operative complications. Both visits will be managed by a clinical research associate through a telephone call;
- A visit 1 year (± 2 weeks) after surgery to evaluate the primary outcome (anatomical and functional success) managed by an independent assessor blinded to the type of surgery;
- A visit 2 years (± 2 weeks) after surgery to evaluate the failure rate of POP surgery with anatomical and functional failures managed by an independent assessor blinded to the type of surgery;
- A visit 3 years (± 2 weeks) after surgery to evaluate the failure rate of POP surgery with anatomical and functional failures managed by an independent assessor blinded to the type of surgery.

Inclusion criteria will be:

- Age ≥ 50 years
- Symptomatic primary prolapse of the anterior vaginal wall with Aa and/or Ba points ≥ 0 according to the POP-Q system
- A positive answer to the question “Do you usually have a bulge or something falling out that you can see or feel in your vaginal area?” (question 3 of the PFDI-20)
- Ability to give informed consent
- Performance status score ≤ 2

Exclusion criteria will be:

- Indication of concomitant myorraphy of levator ani muscles
- History of previous surgical cystocele repair
- Currently evolving gynecologic cancer
- Pregnancy or wish for future pregnancy, lactating woman
- Inability to participate in study follow-up or to provide informed consent or under judicial protection
- Lack of social insurance
- Contraindication of surgical treatment of prolapse
- Inability to read French

### Sample size

The estimated number of required participants is based on the primary outcome. We estimate that the rate of success defined by combined objective and subjective measures of the anterior colporraphy is about 45% at 1 year, with anatomical success defined with Aa and Ba point of 0 [6, 12].

Vaginal patch plastron technique has never been studied with a combined definition of success (anatomical and functional). Its reported anatomical success rate ranges from 93 to 98% while its functional success rate ranges from 74 to 92% [16]. This procedure combines the advantages of techniques used for the management of median and lateral cystoceles. Therefore, we hypothesize that vaginal patch plastron will be more effective than anterior colporraphy regarding the primary outcome. Sample size calculation is based on an expected difference of 20% in the rate of success as defined by the primary outcome. Calculations with alpha = 5% and beta = 20% yielded 96 patients per group. Assuming a 10% rate of lost-to-follow-ups at 1 year, we have planned to include a total of 214 women (107 patients per treatment arm).

To date, 8 centers are participating in the study. We expect to enroll 90 patients per year. With an inclusion period of 30 months, we are hoping for a total of 225 inclusions.

## Discussion

To our knowledge, this study will be the first randomized trial comparing two techniques of vaginal native tissue prolapse surgery with combined anatomical and functional criteria of success. We think that the TAPP trial will help to determine the best native tissue repair technique and thus to contribute to a better standardization of POP surgery.

### Trial status

- Version n° 3 in date of 05/07/2019
- Overall status: recruiting
- Study start: September 11, 2019
- Primary completion: September 11, 2023

## Supplementary information

**Additional file 1.** 1/ POPQ [20]. 2/ PFDI20 [21, 22]. 3/ PGI-I [18]. 4/ CLAVIEN AND DINDO CLASSIFICATION [23]. 5/ PISQ12 [19]

**Additional file 2.** SPIRIT 2013 Checklist: Recommended items to address in a clinical trial protocol and related documents*.

**Additional file 3.** CONSORT CHECKLIST.

## Data Availability

Yes

## Abbreviations

TAPP: Traitement Autologue du ProlaPsus (vaginal native tissues for prolapse surgery)
POP-Q: Pelvic Organ Prolapse Quantification
POP: Pelvic organ prolapse
FDA: Food and Drug Administration
HAS: Haute Autorité de Santé
CNGOF: Collège National des Gynécologues-Obstétriciens Français
PFDI 20: Pelvic Floor Distress Inventory
PGI-I: Patient Global Impression of Improvement
PISQ 12: Pelvic Organ Prolapse/Urinary Incontinence Sexual Questionnaire
