# Supplementary material for: Protocol for a randomized controlled trial to assess two procedures of vaginal native tissue repair for pelvic organ prolapse at the time of the questioning on vaginal prosthesis: the TAPP trial
Source: Trials. 2020 Jul 8;21:624. doi: 10.1186/s13063-020-04512-x (PMC7346411; doi:10.1186/s13063-020-04512-x)
Supplement: Supplementary file 1 — Additional file 1. 1/ POPQ [20]. 2/ PFDI20 [21, 22]. 3/ PGI-I [18]. 4/ CLAVIEN AND DINDO CLASSIFICATION [23]. 5/ PISQ12 [19] [file 13063_2020_4512_MOESM1_ESM.docx]

**FIGURES**

**1/ POPQ** (20)

**
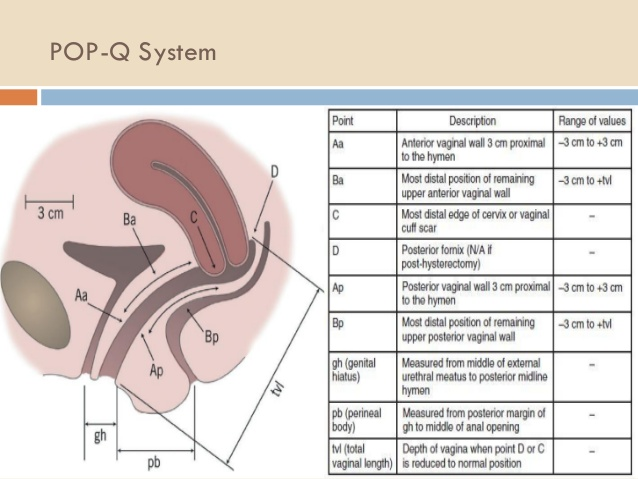
**

**
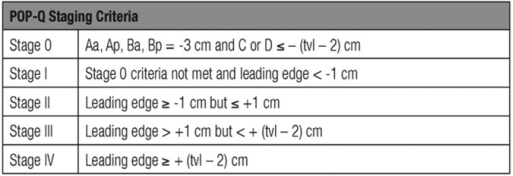
**

**2/ PFDI20** (21,22)


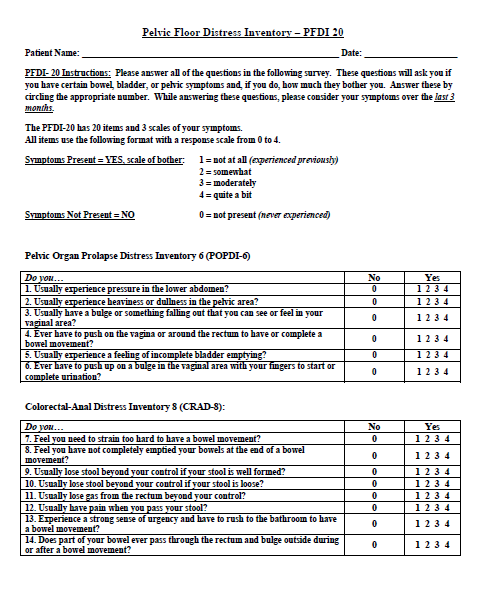


**
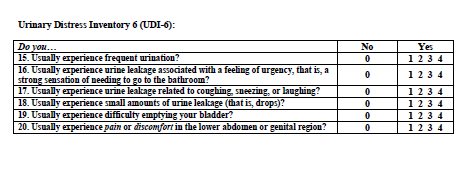
**

**3/ PGI-I** (18)

**Check the number that best describes what your post-operative condition is now, compared with what it was before you had the surgery**:

**1** Very much better

**2**  Much better

**3** A little better

**4** No change

**5** A little worse

**6** Much worse

**7** Very much worse

**4/ CLAVIEN AND DINDO CLASSIFICATION** (23)

The therapy used to correct a specific complication is the basis of this classification in order to rank a complication in an objective and reproducible manner.

It consists of 7 grades (I, II, IIIa, IIIb, IVa, IVb and V). The introduction of the subclasses a and b allows a contraction of the classification into 5 grades (I, II, III, IV and V) depending on the size of the population observed or the of the focus of a study.

Complications that have the potential for long-lasting disability after patient’s discharge (e.g.: paralysis of a voice cord after thyroid surgery) are highlighted in the present classification by a suffix (“d” for disability). This suffix indicates that a follow-up is required to comprehensively evaluate the outcome and related long-term quality of life.

| Grades | Definition |
| --- | --- |
| Grade I | Any deviation from the normal postoperative course without the need for pharmacological treatment or surgical, endoscopic and radiological interventions Allowed therapeutic regimens are: drugs as antiemetics, antipyretics, analgesics, diuretics and electrolytes and physiotherapy. This grade also includes wound infections opened at the bedside. |
| Grade II | Requiring pharmacological treatment with drugs other than such allowed for grade I complications. Blood transfusions and total parenteral nutrition are also included. |
| Grade III | Requiring surgical, endoscopic or radiological intervention |
| - IIIa | Intervention not under general anaesthesia |
| - IIIb | Intervention under general anesthesia |
| Grade IV | Life-threatening complication (including CNS complications)* requiring IC/ICU-management |
| - IVa | single organ dysfunction (including dialysis) |
| - IVb | multiorgan dysfunction |
| Grade V | Death of a patient |

**brain hemorrhage, ischemic stroke, subarrachnoidalbleeding, but excluding transient ischemic attacks (TIA); IC: Intermediate care; ICU: Intensive care unit.*

**5/ PISQ12** (19)

**
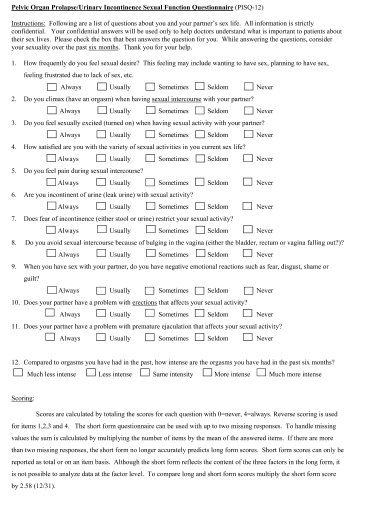
**
