# Supplementary material for: Protocol for a randomized controlled trial to assess two procedures of vaginal native tissue repair for pelvic organ prolapse at the time of the questioning on vaginal prosthesis: the TAPP trial
Source: Trials. 2020 Jul 8;21:624. doi: 10.1186/s13063-020-04512-x (PMC7346411; doi:10.1186/s13063-020-04512-x)
Supplement: Supplementary file 2 — Additional file 2. SPIRIT 2013 Checklist: Recommended items to address in a clinical trial protocol and related documents*. [file 13063_2020_4512_MOESM2_ESM.doc]

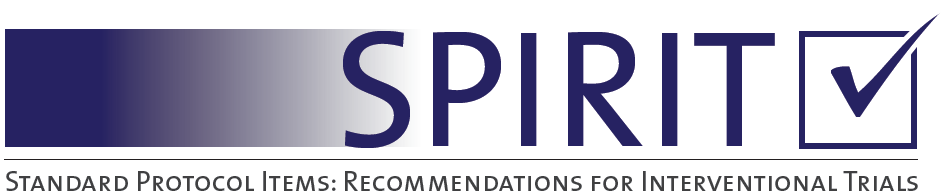


SPIRIT 2013 Checklist: Recommended items to address in a clinical trial protocol and related documents*

| Section/item | ItemNo | Description |
| --- | --- | --- |
| **Administrative information** | | |
| Title  Page 1-2 | 1 | Protocol for a Randomized Controlled Trial to assess two procedures of Vaginal Native Tissue Repair for Pelvic Organ ProlaPse at the time of the questioning on vaginal prosthesis.  The TAPP trial |
| Trial registration  Page 4 | 2a | The trial is registered in the ClinicalTrials.gov registry (NCT03875989). |
|  | 2b | All items from the World Health Organization Trial Registration Data Set |
| Protocol version  Page 14 | 3 | Version n°3.0 du 05/07/2019 |
| Funding  Pages 4-13- | 4 | This research is supported by the French Department of Health (PHRC 2018-A03476-49) and will be conducted with the support of DGOS (PHRC interregional – GIRCI SOHO) |
| Roles and responsibilities | 5a | See pages 1-2 and 4 |
|  | 5b | *CHU LIMOGES* is the sponsor of this research (n°87RI18_0013). 2 avenue Martin Luther King 87042 Limoges Cedex  [drc@chu-limoges.fr](mailto:drc@chu-limoges.fr) |
| Pages 4-14-15 | 5c | *CHU LIMOGES* is the sponsor of this research (n°87RI18_0013).  This research is supported by the French Department of Health (PHRC 2018-A03476-49) and will be conducted with the support of DGOS (PHRC interregional – GIRCI SOHO)  2 avenue Martin Luther King 87042 Limoges Cedex  [drc@chu-limoges.fr](mailto:drc@chu-limoges.fr)  The sponsor and the investigator undertake that this research will be carried out in accordance with European Regulation (EU) No 536/2014 relating to clinical trials of medicinal products for human use and the implementing decree n ° 2016-1537 of November 16, 2016 of the Jardé law relating to research involving the human person (RIPH) no. 2012-300 of March 5, 2012 as well as in accordance with Good Clinical Practice (ICH version 6 of November 15, 2016 and decision of June 24, 2006) and the Declaration of Helsinki (which can be found in its full version on the site http://www.wma.net).  The Limoges University Hospital, promoter of this research, has taken out a civil liability insurance contract with the SHAM in accordance with the provisions of the Public Health Code. The Limoges University Hospital has signed a commitment to comply with this "Reference Methodology".  Any substantial modification, i.e. any modification likely to have a significant impact on the protection of persons, on the conditions of validity and on the results of research, on the quality and safety of the products tested, on the interpretation of the scientific documents which support the conduct of the research or the procedures for conducting it, is subject to a written amendment which is submitted to the promoter; the latter must obtain, prior to its implementation, a favorable opinion from the PPC and, if necessary, an authorization from the ANSM.  All the modifications are validated by the promoter, and by all the research stakeholders concerned by the modification, before submission to the PPC and, if necessary, to the ANSM. This validation may require the meeting of any committee formed for research.  Within one year of the end of the research or its interruption, a final report will be drawn up and signed by the promoter and the investigator. This report will be made available to the competent authority. The promoter will send the research results to the PPC and, if applicable, the ANSM in the form of a summary of the final report within one year of the end of the research.  The publication of the main results mentions the name of the promoter, of all the investigators who included or followed participants in the research, methodologists, biostatisticians and data managers who participated in the research, members of the committee (s) constituted ( s) for research and source of funding. International writing and publishing rules will be taken into account (The Uniform Requirements for Manuscripts of the ICMJE, April 2010). |
|  | 5d | A clinical studies coordinator from CIC 1435 in Limoges will be responsible for:  - the creation of tools allowing an appropriate follow-up of inclusions  - assistance in setting up the study in the centers  - managing the dispatch and monitoring of regulatory documents,  - the management of technical questions from the centers  - drawing up reports concerning its progress,  - communication with participating centers (creation of Newsletters)  The study coordinator will work in close collaboration with the promoter's coordinating investigator and project manager.  At each investigative center, a clinical research technician will be available to:  - ensure data entry in the study base,  - monitor corrective actions requested by the monitor,  - assist the investigators in transmitting the SAEs to the Promoter.  - make phone calls to patients at M4 and M8 post-surgery.  In addition, through the "Territorial Portal" mission carried out by the CIC, clinical research collaborations are already established in the CHs of the Region, and in particular in Brive and Guéret, thus reinforcing the feasibility of the study. Research monitoring at Limoges University Hospital will be provided by a clinical research technician from CIC 1435. He will be responsible, with the coordinating investigator, for:  - research logistics and monitoring (aid for inclusions),  - verification of the update of the observation notebook (request for information additional, corrections, ...),  He will work in accordance with standard operating procedures, in collaboration with the clinical research associate delegated by the sponsor. |
| Introduction |  |  |
| Background and rationale  Pages 5-6-7 | 6a | Pelvic organ prolapse (POP) is usually the result of loss of pelvic support. It is widely accepted that 50% of women after 50 years of age will develop prolapse, evaluated through the POPQ Classification. POP is associated with significant psychological distress and negatively affects quality of life. Cystocele is the main indication for POP surgery (67.7%). Native tissue cystocele repair has been the cornerstone of prolapse surgery especially since the learned societies (Food and Drug Administration, Haute Autorité de Santé, Collège National des Gynécologues-Obstétriciens Français) warned clinicians and patients about serious complications associated with transvaginal meshes. In France, 47.5% of vaginal cystocele repair is based on native tissue repair. Anterior colporraphy and vaginal patch plastron are the most common procedures, with re-intervention rates lower than 4% at one year. However, success rates of native tissue repair are heterogeneous (34.5% to 89%), depending on study design and the definition of outcomes. Furthermore, randomized trials comparing the effectiveness of vaginal native tissue techniques are still lacking. Hence, the choice of the surgical procedure is still not evidence-based and mostly subjective. |
|  | 6b | Among women with POP, cystocele is the most common defect, occurring in 67.7% of case. It is commonly treated by transvaginal repair with native tissue repair (47.5% in France). Sacrocolpopexy using synthetic mesh by laparoscopy is considered the surgical gold standard but this procedure has several contraindications: baseline risk factors for mesh erosion (obesity, smoking, association with hysterectomy) and history of abdominal surgery. The use of the vaginal route, considered as minimally invasive surgery, may thus be indicated in first intention for any patient suffering from symptomatic anterior POP. While transvaginal mesh procedures have been largely studied, current evidence does not support their use as a first-line intervention for anterior compartment prolapse according the CNGOF, HAS and FDA due to significant post-operative morbidity. |
| Objectives  Pages 7-8-9 | 7 | The aim of this study was thus to compare the effectiveness of the vaginal patch plastron versus anterior colporraphy one year after POP surgery, using a combined definition of success based on both anatomical and functional parameters. We estimate that the rate of success defined by combined objective and subjective measures of the anterior colporraphy is about 45% at one year, with anatomical success defined with Aa and Ba point of 0 .  Vaginal patch plastron technique has never been studied with a combined definition of success (anatomical and functional). Its reported anatomical success rate ranges from 93 to 98% while its functional success rate ranges from 74 to 92%. This procedure combines the advantages of techniques used for the management of median and lateral cystoceles. Therefore we hypothesize that vaginal patch plastron will be more effective than anterior colporraphy regarding the primary outcome |
| Trial design  Page 7-8-9 | 8 | Herein we introduce an experimental, multicenter (8 centres) parallel-group randomized controlled trial (1:1) to assess the effectiveness of the vaginal patch plastron at one year postoperatively in comparison with anterior colporraphy through a combined definition of success- anatomical and functional. |
| Methods: Participants, interventions, and outcomes | | |
| Study setting  Pages 7-13 | 9 | To date, 8 centres are participating in the study: CHU of Limoges, CHU of Clermont-Ferrand, CHU of Bordeaux, CHU Purpan of Toulouse, CHU Paule de Viguier of Toulouse, Hospital of Tulle, Hospital of Brive-la-Gaillarde, and Hospital of Guéret. |
| Eligibility criteria  Pages12-13 | 10 | Inclusion criteria will be:  - Age >50 years  - Symptomatic primary prolapse of the anterior vaginal wall with Aa and/or Ba points ≥0 according to the POP-Q system  - A positive answer to the question “Do you usually have a bulge or something falling out that you can see or feel in your vaginal area?” (question 3 of the PFDI-20)  - Ability to give informed consent  - Performance Status score ≤ 2  Exclusion criteria will be:  - Indication of concomitant myorraphy of levator ani muscles  - History of previous surgical cystocele repair.  - Currently evolving gynecologic cancer.  - Pregnancy or wish for future pregnancy, lactating woman.  - Inability to participate in study follow-up or to provide informed consent or under judicial protection.  - Lack of social insurance.  - Contraindication of surgical treatment of prolapse  - Inability to read French |
| Interventions  Page 9-10-11 | 11a | The study will be introduced to all women referred to participating centres for surgical repair of cystocele at the time of their pre-operative visit (screening visit). Patients will be definitively enrolled after giving their informed written consent. |
|  | 11b | Patients will be randomly assigned in a 1:1 ratio in the operating room to undergo anterior colporraphy (reference treatment) or vaginal patch plastron (experimental treatment). |
|  | 11c | Since there is no specific contraindication for any of the two techniques, both surgical treatments can be performed in all patients included in the study. Hence, patients will not be aware of randomization assignment. In contrast, masking for the surgeon with respect to treatment allocated by randomisation will not be feasible. |
|  | 11d | Hysterectomy will not be performed systematically; however, in case of hysterectomy, a sacrospinous ligament fixation or a high McCall culdoplasty will be performed. |
| Outcomes  Pages 7-8-9 | 12 | The primary outcome will be assessed 1 year after surgery by an independent assessor blinded to the allocated treatment arm. It is defined as the success rate of POP surgery defined by a composite of objective and subjective parameters:   - Anatomical success defined by Aa and Ba values <0 in the POP-Q system   AND   - Subjective success through reliable condition-specific quality-of-life questionnaires:   - A negative answer to the question “Do you usually have a bulge or something falling out that you can see or feel in your vaginal area?” (question 3 of the PFDI-20)   AND   - - Range score of PGI-I 1 or 2 (18)   AND  No need for re-intervention (medical or surgical) for recurrence of cystocele Secondary outcomes will comprise:   1. The failure rate of POP surgery defined by a composite of objective and subjective measures evaluated 1 year after surgery:    - Recurrent prolapse defined by Aa and/or Ba values ≥ 0 in POP-Q system   OR   - - Subjective failure through reliable condition-specific quality-of-life questionnaires:     - A positive answer to the question “Do you usually have a bulge or something falling out that you can see or feel in your vaginal area?” (question 3 of the PFDI-20)   OR   - - - A PGI-I score > 2   OR  need for re-intervention (medical or surgical) for recurrence of cystocele This secondary outcome will be assessed 1 year after surgery by an independent assessor blinded to the allocated arm.  2) Rate of post-operative complications according to the Clavien-Dindo classification 45 days after surgery, reported by the patient’s surgeon (not blinded).  3) Sexual function improvement will be evaluated by the difference in PISQ 12 score (19) (condition-specific quality-of-life questionnaire) between the inclusion and one year after surgery for sexually active women.  4) The failure rate of POP surgery defined by a composite of objective and subjective measures evaluated 2 years after surgery  5) The failure rate of POP surgery defined by a composite of objective and subjective measures evaluated 3 years after surgery.  This secondary outcome will be evaluated 1, 2 and 3 years after surgery by an independent assessor blinded to the allocated arm.  The primary outcome and several secondary outcomes (items 1 and 3) will be evaluated according to a double blind protocol. |
| Participant timeline  Page 9 | 13 | The inclusion period will extend over 30 months. The duration of follow-up for each patient will be 42 months. The trial will last 72 months. Patients’ follow-up will comprise:  - a visit 45 days (+- 10 days) after surgery to evaluate the post-operative complications according to the Clavien-Dindo classification (referent surgeon);.  - 2 additional visits at 4 and 8 months (+- 1 week) after surgery to make sure they have not suffered from delayed post-operative complications. Both visits will be managed by a clinical research associate through a telephone call;  - a visit 1 year (+- 2 weeks) after surgery to evaluate the primary outcome (anatomical and functional success) managed by an independent assessor blinded to the type of surgery.  - a visit 2 years (+- 2 weeks) after surgery to evaluate the failure rate of POP surgery with anatomical and functional failures managed by an independent assessor blinded to the type of surgery.  - a visit 3 years (+- 2 weeks) after surgery to evaluate the failure rate of POP surgery with anatomical and functional failures managed by an independent assessor blinded to the type of surgery. |
| Sample size  Page 13 | 14 | The estimated number of required participants is based on the primary outcome. We estimate that the rate of success defined by combined objective and subjective measures of the anterior colporraphy is about 45% at one year, with anatomical success defined with Aa and Ba point of 0 .  Vaginal patch plastron technique has never been studied with a combined definition of success (anatomical and functional). Its reported anatomical success rate ranges from 93 to 98% while its functional success rate ranges from 74 to 92% (. This procedure combines the advantages of techniques used for the management of median and lateral cystoceles. Therefore we hypothesize that vaginal patch plastron will be more effective than anterior colporraphy regarding the primary outcome. Sample size calculation is based on an expected difference of 20% in the rate of success as defined by the primary outcome. Calculations with alpha = 5% and beta = 20% yielded 96 patients per group. Assuming a 10% rate of lost-to-follow-ups at one year, we have planned to include a total of 214 women (107 patients per treatment arm).  To date, 8 centres are participating in the study. We expect to enrol 90 patients per year. With an inclusion period of 30 months, we are hoping for a total of 225 inclusions. |
| Recruitment  P 13 | 15 | Strategies for achieving adequate participant enrolment to reach target sample size   - The centres chosen to include in this study are expert centres for prolapse surgery with a high volume of activity. We estimate the possibility of annual recruitment of patients according to centres chosen at 90 per year. |
| **Methods: Assignment of interventions (for controlled trials)** | | |
| Allocation: |  |  |
| Sequence generation  Page 9 | 16a | The study will be introduced to all women referred to participating centres for surgical repair of cystocele at the time of their pre-operative visit (screening visit). Patients will be definitively enrolled after giving their informed written consent.  Patients will be randomly assigned in a 1:1 ratio in the operating room through computed generator random number to undergo anterior colporraphy (reference treatment) or vaginal patch plastron (experimental treatment).  Since there is no specific contraindication for any of the two techniques, both surgical treatments can be performed in all patients included in the study. Hence, patients will not be aware of randomization assignment. |
| Allocation concealment mechanism  Page 9-10 | 16b | We will randomly assigned participants by a remote web-based randomisation system when the patient will enter into the operating room to one of the two trials:   - Anterior colporraphy (standard treatment) - Or vaginal patch plastron (experimental treatment).   In case of computer problem, a paper randomization list will be available |
| Implementation | 16c | Surgeons will screened, enrolled and assigned randomly patients. |
| Blinding (masking)  Page 9-10 | 17a | Patients will not be aware of randomization assignment. In contrast, masking for the surgeon with respect to treatment allocated by randomisation will not be feasible. The secondary outcome will be evaluated 1, 2 and 3 years after surgery by an independent assessor blinded to the allocated arm.  The primary outcome and several secondary outcomes (items 1 and 3) will be evaluated according to a double blind protocol. |
| Page 9-10 | 17b | The primary outcome and several secondary outcomes (items 1 and 3) will be evaluated according to a double blind protocol (since the patient and his assessor will not be aware of the randomization arm that will have been assigned to the patient)  Hence, patients will not be aware of randomization assignment. In contrast, masking for the surgeon with respect to treatment allocated by randomisation will not be feasible.  The unblinding during the research must remain an exceptional procedure to guarantee the safety of the participant in the study or a third person.  If an investigator considers that he / she needs to know the surgical technique performed on the patient in order to adapt the management in case of emergency and / or absolute necessity for the health of the patient, the investigator may lift the blind. The unblinding will be done online with the access codes of the investigator. If and only if the elimination of online blindness is not possible, the investigator can contact the pharmacy of Limoges University Hospital, which holds the randomization list. |
| **Methods: Data collection, management, and analysis**  *CHU LIMOGES* is the sponsor of this research (n°87RI18_0013).  This research is supported by the French Department of Health (PHRC 2018-A03476-49) and will be conducted with the support of DGOS (PHRC interregional – GIRCI SOHO)  The study protocol was approved by the Human Subjects Protection Review Board (Comité de Protection des Personnes) on May, 16^th^ 2019.  The trial is registered in the ClinicalTrials.gov registry (NCT03875989).  The complete research protocol is accessible in a French version with the description of data collection, management and statistical analysis. | | |
| Data collection methods  Page 9 | 18a | All the information contained in original documents, or in authenticated copies of these documents, relating to clinical examinations, observations or other activities carried out within the framework of research and necessary for the reconstruction and evaluation of the research. The documents in which the source data is saved are called the source documents.  In this study, the source documents will be:  - the medical file,  - quality of life questionnaires completed directly by the patient  - the name sheet for collecting patient data.  This name sheet will be completed specifically for each patient and will make it possible to collect the data necessary for the study, which is not usually indicated in the medical file.  The data will be entered online (via internet) by the investigators after connecting to the database specific to this protocol which is created by the data manager of Limoges University Hospital from Ennov Clinical software. The questionnaires are usually used in the Gynecology Obstetrics Department |
|  | 18b | The participant who wishes to withdraw or withdraw his consent to participate in research (as he is entitled to do at any time), is no longer followed within the framework of the protocol, but must be the subject of the best care possible charge given his state of health and the state of knowledge at the time.  An abandonment is a decision of an included participant to assert his right to interrupt his participation in a research, at any time during the follow-up, without incurring any prejudice from this fact and without having to justify himself .  The investigator should identify the cause of the abandonment if possible and assess whether it is possible to collect the variable on which the primary judgment criterion relates at the time of the abandonment. Research abandonments must be notified promptly to the promoter's legal representative in France by fax. The reasons and the date of abandonment must be documented in the observation book.  In the event of abandonment before performing the surgery, the investigator will not perform an analysis of the data collected as part of the research.  In the event of abandonment after randomization and surgery, the investigator will propose to the patient to resume classic monitoring and the data collected in the course of the research will be kept for statistical analysis in accordance with article 17 of the GDPR.  Withdrawal of consent is a participant's decision to reverse their decision to participate in research and to assert their right to withdraw their informed consent, at any time during the follow-up and without incurring any prejudice of this fact and without having to be justified.  When a participant withdraws or withdraws consent to participate in research, the investigator must contact the promoter's legal representative in France. In accordance with art. L1122-1 of the CSP, the data already collected will remain acquired by the promoter. |
| Data management | 19 | Plans for data entry, coding, security, and storage, including any related processes to promote data quality (eg, double data entry; range checks for data values). Reference to where details of data management procedures can be found, if not in the protocol  Ennov Clinical software complies with the 21 CFR Part 11 standard of the Food and Drug Administration, as well as the standard concerning the security of computerized systems. The data can be printed and locked at any time, according to the rights of the user. The data entered is saved in real time. A daily backup of the database is done according to the internal procedure set up at Limoges University Hospital.  Data validation:  The consistency tests will be configured and executed either directly under Ennov Clinical via the CSTest module, or under SAS after data extraction from the database via CSExport.  Data to meet the main objective is mandatory. The use of a missing data code (ND, NK, NA ...) may be tolerated on this data, subject to a justification for the non-obtaining of this data, motivated by the investigator.  Pre-tests running on data entry can be set up, in agreement with the investigator and his team.  A list of authorized obvious corrections will also be defined with the investigator.  All of the consistency tests will be included in the Data Management Plan (DMP), a document which will be archived with the rest of the study documents.  All consistency checks returning a discrepancy of data, outside the framework of any obvious corrections and tolerated missing data code, will give rise to the issue of queries. Queries will be transmitted online and must be corrected by the investigator or a person designated by him in the task delegation list.  The emission of queries may also be made by a CRA monitor designated by the promoter. These queries must be corrected by the investigator or a person he will designate from the task delegation list.  All of the data and queries will have to be reviewed by the center’s investigator who will give his agreement on the quality of the data collected by electronically signing the CRFs of his center at the level of the general progress table under CSOnline.  The database could be frozen after correction of all queries and signatures of CRFs and blind data review by a data review committee, composed of the principal investigator, the methodologist, the biostatistician and the vigilant (if necessary), ARC monitor, project manager and data-manager.  A basic freezing form must attest to the authorization of the principal investigator to no longer modify the data. |
| Statistical methods  Page 13 | 20a | Comparisons will be made between two groups of randomization in an intention-to-treat analysis.  Statistical analyses will be computed with the SAS V9.3 (SAS Institute Cary, NC) software. The significant threshold will be fixed at 0,05 for all the analyses which will follow the revised CONSORT statements of 2010. A flow chart will also be presented. Quantitative variables will be presented with the mean, the standard deviation, the minimum, the maximum and the median with the interquartile interval. Qualitative variables will be presented with the number and percentages. Data will be described globally and by randomization group.  The main analysis will be a Pearson Chi² test or a Fisher exact test according to expected numbers. The same analysis will be computed for each component of the main outcome and for the secondary analysis which will compare the proportion of (i) failed procedures at 1 year, 2 year and 3 year of follow-up and (ii) post-surgery complications between the two randomized groups. The evolution of the sexual life from baseline to 1 year of follow up will be compared between the two randomized groups with a Student test or a Mann-Whitney test according to the distribution of the variable. |
|  | 20b | All data will be analysed using SAS® 9.3 (SAS Institute Cary, NC) and a *p* value < 0.05 will be considered statistically significant.  Not Applicable |
|  | 20c | A flowchart will be presented.  Statistical analyses will be computed in the intent to treat population which will include all randomized patients. A complementary analyse of the primary analyse will be realised on the per-protocol population which will exclude all patients who will not follow the protocol. |
| **Methods: Monitoring** | | |
| Data monitoring | 21a | Composition of data monitoring committee (DMC); summary of its role and reporting structure; statement of whether it is independent from the sponsor and competing interests; and reference to where further details about its charter can be found, if not in the protocol. Alternatively, an explanation of why a DMC is not needed  The two surgical techniques that we wish to study are the most used techniques and proposed in first intention to the patients likely to profit from a surgical treatment of a cystocele, with low and acceptable rates of complications per and postoperative and the absence risks of complications related to the use of prosthetic equipment. Therefore, it is not considered necessary to set up an Independent Monitoring Committee. |
|  | 21b | Description of any interim analyses and stopping guidelines, including who will have access to these interim results and make the final decision to terminate the trial  Not interim analysis. Clinical research may be stopped early by the sponsor. The latter justifies his judgment in the statement he makes to the competent authority and the ethics committee.  The statistical analyzes will be carried out by the Cebimer of the Limoges University Hospital using SAS® 9.3 software (SAS Institute Cary, NC). The degree of statistical significance used for all analyzes will be set at 0.05. The analyzes will be carried out with the intention of treating. The analyzes will be conducted and presented in accordance with the CONSORT Statement recommendations revised in 2010 [17]  A flow diagram will be presented. The alpha risk is set at 5%  Missing data will not be replaced  The quantitative variables will be described according to the mean ± standard deviation or the median and the interquartile range.  Qualitative variables will be described by numbers and percentages. The variables will be described globally and then by randomization group in order to check the initial comparability of the two groups.  The main analysis will correspond to the comparison of the proportion of success (primary outcome measure) between the two randomization groups using the Chi2 test or Fisher's exact test (depending on compliance with the conditions of application of the Chi2 test).  The various components of the primary outcome (anatomical success, functional success and lack of indication of a new treatment) will also be described and compared between the groups as part of an additional analysis.  Secondary analysis n ° 1 will correspond to the comparison of the proportions of failure between the two randomization groups using the Chi2 test or Fisher's exact test (depending on compliance with the conditions of application of the Chi2 test). The different components of the secondary judgment criterion No. 1 will also be described and compared between the groups as part of a complementary analysis.  Secondary analysis n ° 2 will correspond to a description of all postoperative complications and to a comparison of postoperative complication rates using the statistical techniques described above.  The secondary analysis n ° 3 corresponding to the evaluation of the improvement of the sexual life will use the student t test or Mann Whitney test if the conditions of application of the student test are not verified. The variable compared between the two groups will be the difference in the PISQ12 score between the date of inclusion and 1 year. |
| Harms | 22 | Plans for collecting, assessing, reporting, and managing solicited and spontaneously reported adverse events and other unintended effects of trial interventions or trial conduct  In the case of research involving the human person with minimal risks and constraints, the usual vigilance of care will apply for this study. Adverse events / adverse effects / incidents will be declared to the different circuits  sanitary vigilance applicable to each product or practice concerned (care vigilance, pharmacovigilance, materiovigilance, haemovigilance, cosmetovigilance ...) in accordance with the current regulations.  The two surgical techniques that we wish to study are the most used and proposed as first-line techniques for patients likely to benefit from surgical treatment of a cystocele, with low and acceptable rates of per and postoperative complications and the absence risks of complications related to the use of prosthetic equipment. Therefore, it is not considered necessary to set up an Independent Supervisory Committee. |
| Auditing | 23 | Frequency and procedures for auditing trial conduct, if any, and whether the process will be independent from investigators and the sponsor  A Clinical Research Assistant appointed by the sponsor regularly visits each site investigator, when setting up the research, one or more times during the research according to the rhythm of the inclusions and at the end of the research. During these visits, the elements controlled by the CRA depend on the monitoring level defined in the monitoring plan which is based on  on the risk.  An audit may be carried out at any time by persons appointed by the promoter and independent of the persons conducting the research. It aims to verify the safety of participants and respect for their rights, compliance with applicable regulations and the reliability of data  An inspection can also be carried out by a competent authority (ANSM for France or EMA in the context of a European test for example).  The audit, as well as the inspection, can be applied at all stages of research, from the development of the protocol to the publication of the results and the classification of the data used or produced within the framework of the research.  The investigators agree to comply with the requirements of the promoter with regard to an audit and with the competent authority for a research inspection. |
| **Ethics and dissemination** | | |
| Research ethics approval  Page 14-15 | 24 | Plans for seeking research ethics committee/institutional review board (REC/IRB) approval  The protocol received the favorable opinion of the Ethics Committee named “Comité de Protection des Personnes Ile De France VIII” on June 18^th^ 2019. |
| Protocol amendments | 25 | Plans for communicating important protocol modifications (eg, changes to eligibility criteria, outcomes, analyses) to relevant parties (eg, investigators, REC/IRBs, trial participants, trial registries, journals, regulators)  Each important protocol modification is submitted for opinion to the Ethics Committee. It is necessary for its implementation.  Any substantial modification, i.e. any modification likely to have a significant impact on the protection of persons, on the conditions of validity and on the results of research, on the quality and safety of the products tested, on the interpretation of the scientific documents which support the conduct of the research or the procedures for conducting it, is subject to a written amendment which is submitted to the promoter; the latter must obtain, prior to its implementation, a favorable opinion from the PPC and, if necessary, an authorization from the ANSM.  Insubstantial changes, i.e. those that do not have a significant impact on any aspect of the research, are communicated to the PPC for information.  All the modifications are validated by the promoter, and by all the research stakeholders concerned by the modification, before submission to the PPC and, if necessary, to the ANSM. This validation may require the meeting of any committee formed for research. .  All modifications to the protocol must be brought to the attention of all investigators participating in the research. The investigators agree to respect the content.  Any modification which modifies the assumption of responsibility of the participants or the benefits, risks and constraints of the research is the subject of a new information note and a new consent form, the collection of which follows the same procedure as that mentioned above. . |
| Consent or assent  Page 9 | 26a | Who will obtain informed consent or assent from potential trial participants or authorised surrogates, and how (see Item 32)  During the pre-inclusion / inclusion visit, the study investigators informs the patient and responds to all questions concerning the purpose, the nature of the constraints, the foreseeable risks and the expected benefits of the research. It also specifies the rights of the patient as part of a researches and verifies the eligibility criteria. Prior to any research-related examination, the study investigators will collect free, informed consent and wrote of the patient. |
|  | 26b | Additional consent provisions for collection and use of participant data and biological specimens in ancillary studies, if applicable  On the consent form, participants will be asked if they agree to use of their data should they choose to withdraw from the trial. Participants will also be asked for permission for the research team to share relevant data with people from the Universities taking part in the research or from regulatory authorities, where relevant. This trial does not involve collecting biological specimens for storage. |
| Confidentiality | 27 | How personal information about potential and enrolled participants will be collected, shared, and maintained in order to protect confidentiality before, during, and after the trial  In accordance with the legislative provisions in force, persons having direct access to source data will take all necessary precautions to ensure the confidentiality of the data information about experimental drugs, research, people who and, in particular, as regards their identity and the results obtained. These persons, like the investigators themselves, are subject to professional secrecy.  During or after the research, the data collected on the people who are  sent to the sponsor by the investigators will be made anonymous. In no case should they show in clear the names of the persons concerned or their address.  Only the first letters of the patient's name and first name will be recorded, accompanied a coded number specific to the research indicating the order of inclusion of the patients.  The promoter will ensure that each person who lends himself to research has given his written agreement for access to individual data concerning him and strictly necessary for the quality control of research. |
| Declaration of interests | 28 | Financial and other competing interests for principal investigators for the overall trial and each study site  No conflict of interest declared |
| Access to data | 29 | Statement of who will have access to the final trial dataset, and disclosure of contractual agreements that limit such access for investigators  Only a few employees at Limoges University Hospital will have access to data.  Any data required to support the protocol can be supplied on request |
| Ancillary and post-trial care | 30 | Provisions, if any, for ancillary and post-trial care, and for compensation to those who suffer harm from trial participation  There is no anticipated harm and compensation for trial participation |
| Dissemination policy | 31a | Plans for investigators and sponsor to communicate trial results to participants, healthcare professionals, the public, and other relevant groups (eg, via publication, reporting in results databases, or other data sharing arrangements), including any publication restrictions  In accordance with the law n ° 2002-303 of March 4th, 2002, the participants are informed, at their request, overall results of the research. The results will be made public in the form of articles in scientific journals.  The analysis of data provided by the investigative centers is carried out by CEBIMER. This analysis gives rise to a written report which is submitted to the promoter, who will forward it to the Personal Protection Committee and to the competent authority.  Any written or oral communication of the research results must receive the prior agreement of the coordinating investigator and, if applicable, of any committee set up for the research.  The coordinating / principal investigator undertakes to make the research results both negative and inconclusive as positive, available to the public.  The publication of the main results mentions the name of the promoter, of all the investigators who included or followed participants in the research, methodologists, biostatisticians and data managers who participated in the research, members of the committee (s) constituted ( s) for research and source of funding. International writing and publishing rules will be taken into account (The Uniform Requirements for Manuscripts of the ICMJE, April 2010). |
|  | 31b | Authorship eligibility guidelines and any intended use of professional writers |
| Page 1-2 |  | The publication of the main results mentions the name of the sponsor, all investigators having included or followed research participants, methodologists, biostatisticians and data directors who have trained for research, members of the committee (s) established for the research and the source of funding. Account will be taken of international rules of writing and publication (Uniform Requirements for ICMJE Manuscripts, April 2010).  All named authors adhere to the authorship guidelines of Trials. All authors have agreed to publication. |
| Page 1-2 | 31c | Plans, if any, for granting public access to the full protocol, participant-level dataset, and statistical code  This search is registered at <http://clinicaltrials.gov/> under number NCT03875989.  Only a few employees at Limoges University Hospital will have access to data" please be consistent. The datasets analysed during the current study are available from the corresponding author on reasonable request. |
| **Appendices** |  |  |
| Informed consent materials  Page 9 | 32 | Model consent form and other related documentation given to participants and authorised surrogates  These are available from the corresponding author on request. |
| Biological specimens | 33 | Plans for collection, laboratory evaluation, and storage of biological specimens for genetic or molecular analysis in the current trial and for future use in ancillary studies, if applicable  This trial does not involve collecting biological specimens for storage. |

*It is strongly recommended that this checklist be read in conjunction with the SPIRIT 2013 Explanation & Elaboration for important clarification on the items. Amendments to the protocol should be tracked and dated. The SPIRIT checklist is copyrighted by the SPIRIT Group under the Creative Commons “[Attribution-NonCommercial-NoDerivs 3.0 Unported](http://www.creativecommons.org/licenses/by-nc-nd/3.0/)” license.
