# Supplementary material for: Protocol for a randomized controlled trial to assess two procedures of vaginal native tissue repair for pelvic organ prolapse at the time of the questioning on vaginal prosthesis: the TAPP trial
Source: Trials. 2020 Jul 8;21:624. doi: 10.1186/s13063-020-04512-x (PMC7346411; doi:10.1186/s13063-020-04512-x)

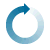


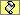
[
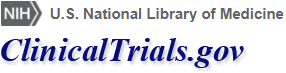
](https://clinicaltrials.gov/ct2/home)


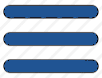


**×**

- [Find Studies
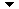
](https://clinicaltrials.gov/ct2/search/index)
  - [New Search](https://clinicaltrials.gov/ct2/home)
  - [Advanced Search](https://clinicaltrials.gov/ct2/search/advanced)
  - [See Studies by Topic](https://clinicaltrials.gov/ct2/search/browse?brwse=cond_cat)
  - [See Studies on Map](https://clinicaltrials.gov/ct2/search/map)
  - [How to Search](https://clinicaltrials.gov/ct2/help/how-find/index)
  - [How to Use Search Results](https://clinicaltrials.gov/ct2/help/how-use-search-results)
  - [How to Find Results of Studies](https://clinicaltrials.gov/ct2/help/how-find/find-study-results)
  - [How to Read a Study Record](https://clinicaltrials.gov/ct2/help/how-read-study)
- [About Studies
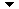
](https://clinicaltrials.gov/ct2/about-studies)
  - [Learn About Studies](https://clinicaltrials.gov/ct2/about-studies/learn)
  - [Other Sites About Studies](https://clinicaltrials.gov/ct2/about-studies/other-sites)
  - [Glossary of Common Site Terms](https://clinicaltrials.gov/ct2/about-studies/glossary)
- [Submit Studies
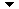
](https://clinicaltrials.gov/ct2/manage-recs)
  - [Why Should I Register and Submit Results?](https://clinicaltrials.gov/ct2/manage-recs/background)
  - [FDAAA 801 and the Final Rule](https://clinicaltrials.gov/ct2/manage-recs/fdaaa)
  - [How to Apply for an Account](https://clinicaltrials.gov/ct2/manage-recs/how-apply)
  - [How to Register Your Study](https://clinicaltrials.gov/ct2/manage-recs/how-register)
  - [How to Edit Your Study Record](https://clinicaltrials.gov/ct2/manage-recs/how-edit)
  - [How to Submit Your Results](https://clinicaltrials.gov/ct2/manage-recs/how-report)
  - [Frequently Asked Questions](https://clinicaltrials.gov/ct2/manage-recs/faq)
  - [Support Materials](https://clinicaltrials.gov/ct2/manage-recs/resources)
  - [Training Materials](https://clinicaltrials.gov/ct2/manage-recs/present)
- [Resources
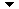
](https://clinicaltrials.gov/ct2/resources)
  - [Selected Publications](https://clinicaltrials.gov/ct2/resources/pubs)
  - [Clinical Alerts and Advisories](https://clinicaltrials.gov/ct2/resources/alert)
  - [RSS Feeds](https://clinicaltrials.gov/ct2/resources/rss)
  - [Trends, Charts, and Maps](https://clinicaltrials.gov/ct2/resources/trends)
  - [Downloading Content for Analysis](https://clinicaltrials.gov/ct2/resources/download)
- [About Site
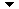
](https://clinicaltrials.gov/ct2/about-site)
  - [What's New](https://clinicaltrials.gov/ct2/about-site/new)
  - [ClinicalTrials.gov Background](https://clinicaltrials.gov/ct2/about-site/background)
  - [About the Results Database](https://clinicaltrials.gov/ct2/about-site/results)
  - [History, Policies, and Laws](https://clinicaltrials.gov/ct2/about-site/history)
  - [Media/Press Resources](https://clinicaltrials.gov/ct2/about-site/for-media)
  - [Linking to This Site](https://clinicaltrials.gov/ct2/about-site/link-to)
  - [Terms and Conditions](https://clinicaltrials.gov/ct2/about-site/terms-conditions)
  - [Disclaimer](https://clinicaltrials.gov/ct2/about-site/disclaimer)

Haut du formulaire

**
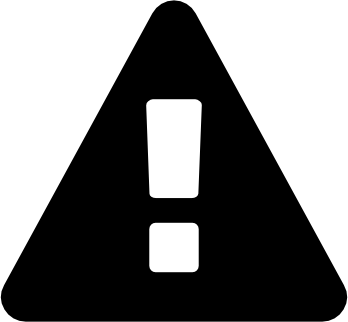
Warning**

**You have reached the maximum number of saved studies (100).**

**Please remove one or more studies before adding more.**

Bas du formulaire

Vaginal Native Tissues Repair for Pelvic Organ Prolapse (TAPP)

|  | The safety and scientific validity of this study is the responsibility of the study sponsor and investigators. Listing a study does not mean it has been evaluated by the U.S. Federal Government. [Know the risks and potential benefits](https://clinicaltrials.gov/ct2/about-studies/learn#Considerations) of clinical studies and talk to your health care provider before participating. Read our [disclaimer](https://clinicaltrials.gov/ct2/about-site/disclaimer) for details. |
| --- | --- |

| ClinicalTrials.gov Identifier: NCT03875989 |
| --- |
| Recruitment Status : Not yet recruiting  First Posted : March 15, 2019  Last Update Posted : July 8, 2019 |

**Sponsor:**

University Hospital, Limoges

**Information provided by (Responsible Party):**

University Hospital, Limoges

**Study Description**

The aim of the study is to assess at one year the effectiveness of the vaginal patch plastron in comparison of the anterior colporraphy through a combined definition of success: anatomic and functional.

| **Condition or disease** | **Intervention/treatment** | **Phase** |
| --- | --- | --- |
| Prolapse, Vaginal | Procedure: Anterior colporraphyProcedure: vaginal patch plastron | Not Applicable |

Detailed Description:

Pelvic organ prolapse is usually the result of loss of pelvic support. It is widely accepted that 50% of women after 50 years old will develop prolapse, evaluated through the POPQ Classification . Pelvic organ prolapse cause significant psychological distress and negatively affect quality of life. Among the surgery for prolapse, the cystocele cureis is the most frequent (67.7%). Native tissue cystocele repairs is the cornerstone of prolapse surgery especially since the learned societies (Food and Drug Administration, Haute Autorité de Santé, Collège National des Gynécologues-Obstétriciens Français) warned clinicians and patients about serious mesh related complications. In France, 41.5% of vaginal cystocele repair are with native tissue. The main surgeries are anterior colporraphy and vaginal patch plastron, used in routine in our center with re-intervention rates less than 4% at one year. Rate of success of native tissue cystocele repair are heterogeneous, depending on the design of studies and definition of outcomes. A prospective study find a success rate at 35% of the anterior colporraphy based on a combined definition, anatomic and functional as recommended recently. However the definition of anatomic was strict (POPQ<2), while it seems that the best definition of anatomic success is "no prolapse among the hymen", that is to say aAa and Ba points <0.

We think that the vaginal patch plastron will have a better anatomic and functional success comparatively to the anterior colporraphy as it corrects median cystoceles by a vaginal strip as well as lateral cystoceles by the bilateral paravaginal suspension.

The description of the two surgeries will be standardized between all the surgeons. We will compare the anterior colporraphy consisting in bladder median support by retensioning Halban fascia with colpectomy to the vaginal patch plastron consisting in making a vaginal strip attached to the bladder combined with suspension by fixation of the vaginal strip to the tendinous arch of the pelvic fascia.

Patients will be blind of their surgery. They will have a follow-up visit 45 days after the surgery to evaluate the post- operative complications according to the Clavien-Dindo classification. They will have phone call at 4 and 8 months after the surgery to make sure they've not suffered for complications. At last, they will have a follow-up visit 1 year after the surgery by an independent assessor blind of the surgery to evaluate the primary outcome (anatomic and functional success).

**Study Design**

|  |  |
| --- | --- |
| Study Type : | Interventional  (Clinical Trial) |
| Estimated Enrollment : | 214 participants |
| Allocation: | Randomized |
| Intervention Model: | Parallel Assignment |
| Intervention Model Description: | Experimental, parallel-group randomised controlled trial (1:1), multicentric. The primary outcome and the secondary outcome 1 and 3 will be evaluated in double blind; only the secondary outcome 2 will not be evaluated in blind. |
| Masking: | Double (Participant, Investigator) |
| Primary Purpose: | Treatment |
| Official Title: | Vaginal Native Tissues Repair for Pelvic Organ Prolapse |
| Estimated Study Start Date : | September 1, 2019 |
| Estimated Primary Completion Date : | September 1, 2025 |
| Estimated Study Completion Date : | September 1, 2025 |

**Arms and Interventions**

| **Arm** | **Intervention/treatment** |
| --- | --- |
| Active Comparator: Arm A | Procedure: Anterior colporraphy  It will be delimitate a rectangular vaginal strip which will be isolated from the anterior colpocele. The superior edge of the strip is placed 2 cm from the urethral orifice. After lateral vesico-vaginal dissection, the paravesical fossae will be wide opened to repair the tendinous arches. The vaginal plastron will be fixed to the tendinous arch of the pelvic fascia by 3 lateral stitches (anterior/ lateral/ posterior) on each side of the plastron. After, the plastron will be tensioning and the cystocele will be suspended. The closure of the vaginal wall will end the procedure. |
| Experimental: Arm B | Procedure: vaginal patch plastron  It will be make a midline incision of the anterior vaginal wall from the urethrovesical junction to the vaginal apex or anterior fornix. The vaginal epithelium will be separated from the underlying fibromuscular layer (Halban Fascia) after the midline incision. Midline plication of the fibromuscular layer will be obtained by interrupted horizontal stiches. The closure of the vaginal wall will end the procedure. |

**Outcome Measures**

Primary Outcome Measures :

1. Rate of the prolapse surgery [ Time Frame: 1 year ]

The success rate of the prolapse surgery defined by a composite of objective and subjective measures:

- - Anatomic success defined by Aa and Ba values <0 in Pelvic Organ Prolapse Quantification System (POP-Q) AND
  - Subjective success through reliable condition-specific quality-of-life questionnaires:
    - A negative response to the question "Do you usually have a bulge or something falling out that you can see or feel in your vaginal area?" (question 3 of the Pelvic Floor Distress Inventory (PFDI-20)) AND
    - Range score of Patient Global Impression of Improvement (PGI-I) 1 or 2 AND
  - No need for other treatment for prolapse (surgical nor medical)

Secondary Outcome Measures :

1. Rate of the prolapse surgery [ Time Frame: 1 year ]

The failure rate of the prolapse surgery defined by a composite of objective and subjective measures:

- - Recurrent prolapse defined by Aa and/or Ba values > or= 0 in POP-Q OR
  - Subjective failure through reliable condition-specific quality-of-life questionnaires:
    - A positive response to the question "Do you usually have a bulge or something falling out that you can see or feel in your vaginal area?" (question 3 of the PFDI-20) OR
    - A PGI-I score > 2 OR
  - Need of a new treatment for prolapse (surgical or medical)

1. Rate of post-operative complications [ Time Frame: 45 Days ]

Rate of post-operative complications according to the Clavien-Dindo classification 45 days after the surgery by the patient's surgeon (not blinded)

1. Sexual function [ Time Frame: 1 year ]

The sexual function improvement will be evaluated by the difference in PISQ 12 score (condition-specific quality-of-life questionnaire) between the inclusion and one year after the surgery for sexually active women

1. Rate of the prolapse surgery [ Time Frame: 2 years ]

The failure rate of the prolapse surgery defined by a composite of objective and subjective measures:

- - Recurrent prolapse defined by Aa and/or Ba values > or= 0 in POP-Q OR
  - Subjective failure through reliable condition-specific quality-of-life questionnaires:
    - A positive response to the question "Do you usually have a bulge or something falling out that you can see or feel in your vaginal area?" (question 3 of the PFDI-20) OR
    - A PGI-I score > 2 OR
  - Need of a new treatment for prolapse (surgical or medical)

1. Rate of the prolapse surgery [ Time Frame: 3 years ]

The failure rate of the prolapse surgery defined by a composite of objective and subjective measures:

- - Recurrent prolapse defined by Aa and/or Ba values > or= 0 in POP-Q OR
  - Subjective failure through reliable condition-specific quality-of-life questionnaires:
    - A positive response to the question "Do you usually have a bulge or something falling out that you can see or feel in your vaginal area?" (question 3 of the PFDI-20) OR
    - A PGI-I score > 2 OR
  - Need of a new treatment for prolapse (surgical or medical)

**Eligibility Criteria**

*Choosing to participate in a study is an important personal decision. Talk with your doctor and family members or friends about deciding to join a study. To learn more about this study, you or your doctor may contact the study research staff using the contacts provided below. For general information,* [*Learn About Clinical Studies.*](https://clinicaltrials.gov/ct2/about-studies/learn)

| Layout table for eligibility information | |
| --- | --- |
|  |  |
| Ages Eligible for Study: | 50 Years and older   (Adult, Older Adult) |
| Sexes Eligible for Study: | Female |
| Accepts Healthy Volunteers: | No |

**Criteria**

Inclusion Criteria:

- Patient at 50 years of age or older
- Symptomatic primary prolapse of the anterior vaginal wall defined by Aa and/or Ba points ≥0 according to the POP-Q system
- A positive response to the question "Do you usually have a bulge or something falling out that you can see or feel in your vaginal area?" (question 3 of the PFDI-20)
- Able to give informed consent
- Performans Status score ≤ 2

Exclusion Criteria:

- Patient with need for surgical treatment for myorraphy of levator ani muscles
- Patient with previous surgical cystocele repair.
- Patient with evolving gynaecologic cancer.
- Pregnancy or wish for future pregnancy, lactating woman.
- Inability to participate in study follow-up or to provide informed consent.
- Lack of social insurance .

**Contacts and Locations**

*To learn more about this study, you or your doctor may contact the study research staff using the contact information provided by the sponsor.*

*Please refer to this study by its ClinicalTrials.gov identifier (NCT number):* ***NCT03875989***

Locations

| Layout table for location information | |
| --- | --- |
|  |  |
| **France** | |
| CHU de Bordeaux | **Not yet recruiting** |
| Bordeaux, France, 33000 | |
| Contact: Claude HOCKE, MD | |
| Principal Investigator: Claude HOCKE, MD | |
| Sub-Investigator: Mathilde CORET, MD | |
| Sub-Investigator: Jean-Luc BRUN, MD | |
| Sub-Investigator: Géraldine CHAUVIN, MD | |
| CH de Brive | **Not yet recruiting** |
| Brive-la-Gaillarde, France, 19100 | |
| Contact: Christelle MESNARD, MD | |
| Principal Investigator: Christelle MESNARD, MD | |
| Sub-Investigator: Tristan GAUTHIER, MD | |
| Sub-Investigator: Dimitrios HARISIS, MD | |
| CHU de Clermont Ferrand | **Not yet recruiting** |
| Clermont-Ferrand, France, 63100 | |
| Contact: Sandrine CAMPAGNE, MD | |
| Principal Investigator: Sandrine CAMPAGNE, MD | |
| CH de Gueret | **Not yet recruiting** |
| Guéret, France, 23000 | |
| Contact: Yves AUBARD, MD | |
| Principal Investigator: Yves AUBARD, MD | |
| Sub-Investigator: Aymeline LACORRE, MD | |
| CHU de Limoges | **Not yet recruiting** |
| Limoges, France, 87000 | |
| Contact: Aymeline LACORRE, MD       [aymeline.lacorre@chu-limoges.fr](mailto:aymeline.lacorre@chu-limoges.fr?subject=NCT03875989, 87RI18_0013 (TAPP), Vaginal Native Tissues Repair for Pelvic Organ Prolapse) | |
| Contact: Tristan GAUTHIER, MD       [tristan.gauthier@chu-limoges.fr](mailto:tristan.gauthier@chu-limoges.fr?subject=NCT03875989, 87RI18_0013 (TAPP), Vaginal Native Tissues Repair for Pelvic Organ Prolapse) | |
| Principal Investigator: Aymeline LACORRE, MD | |
| Sub-Investigator: Tristan GAUTHIER, MD | |
| Sub-Investigator: Yves AUBARD, MD | |
| Sub-Investigator: Hugues CALY, MD | |
| Sub-Investigator: Maxime LEGROS, MD | |
| Sub-Investigator: François MARGUERITTE, MD | |
| CHU de Toulouse - Paule de Viguier | **Not yet recruiting** |
| Toulouse, France, 31059 | |
| Contact: Fabien VIDAL, MD | |
| Principal Investigator: Fabien VIDAL, MD | |
| Sub-Investigator: Yann TANGUY LE GAC, MD | |
| Sub-Investigator: Géraldine CARTRON, MD | |
| Sub-Investigator: Martin BAUJAT, MD | |
| CHU de Toulouse - Rangueil | **Not yet recruiting** |
| Toulouse, France, 31400 | |
| Contact: Elodie CHANTALAT, MD | |
| Principal Investigator: Elodie CHANTALAT, MD | |
| Sub-Investigator: Jacques RIMAILHO, MD | |
| Sub-Investigator: Stéphanie MOTTON, MD | |
| Sub-Investigator: Marc SOULE-THOLY, MD | |
| CH de Tulle | **Not yet recruiting** |
| Tulle, France, 19000 | |
| Contact: François SIEGERTH, MD | |

**Sponsors and Collaborators**

University Hospital, Limoges

| Layout table for additonal information | |
| --- | --- |
|  |  |
| Responsible Party: | University Hospital, Limoges |
| ClinicalTrials.gov Identifier: | [NCT03875989](https://clinicaltrials.gov/show/NCT03875989)     [History of Changes](https://clinicaltrials.gov/ct2/archive/NCT03875989) |
| Other Study ID Numbers: | 87RI18_0013 (TAPP) |
| First Posted: | March 15, 2019    [Key Record Dates](https://clinicaltrials.gov/ct2/keydates/NCT03875989) |
| Last Update Posted: | July 8, 2019 |
| Last Verified: | June 2019 |
| Individual Participant Data (IPD) Sharing Statement: | |
| Plan to Share IPD: | No |

| Layout table for additional information | |  |
| --- | --- | --- |
|  |  |  |
| Studies a U.S. FDA-regulated Drug Product: | | No |
| Studies a U.S. FDA-regulated Device Product: | | No |

Keywords provided by University Hospital, Limoges:

|  |  |
| --- | --- |
| Prolapse Vaginal native tissues repair Pelvic organ |  |

Additional relevant MeSH terms:

| Layout table for MeSH terms | |
| --- | --- |
|  |  |
| Prolapse Pelvic Organ Prolapse Uterine Prolapse | Pathological Conditions, Anatomical Uterine Diseases Genital Diseases, Female |


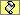

Supplement: Supplementary file 3 — Additional file 3. CONSORT CHECKLIST. [file 13063_2020_4512_MOESM3_ESM.doc]
